# “I Found Out I was Pregnant, and I Started Feeling Stressed”: A Longitudinal Qualitative Perspective of Mental Health Experiences Among Perinatal Women Living with HIV

**DOI:** 10.1007/s10461-021-03283-z

**Published:** 2021-05-16

**Authors:** Emily L. Tuthill, Ann E. Maltby, Belinda C. Odhiambo, Eliud Akama, Jennifer A. Pellowski, Craig R. Cohen, Sheri D. Weiser, Amy A. Conroy

**Affiliations:** 1 Department of Community Health Systems, School of Nursing, University of California, 2 Koret Way, San Francisco, CA 94143 USA; 2 Global Programs, University of California, San Francisco, CA USA; 3 Kenya Medical Research Institute- Center for Microbiology Research, Nairobi, Kenya; 4 Department of Behavioral and Social Sciences, Brown University School of Public Health, Providence, RI USA; 5 International Health Institute, Brown University School of Public Health, Providence, RI USA; 6 Department of Epidemiology and Biostatistics, University of Cape Town, Cape Town, South Africa; 7 Department of Obstetrics, Gynecology and Reproductive Sciences, University of California San Francisco, San Francisco, CA USA; 8 Division of HIV, Infectious Disease, and Global Medicine, Department of Medicine, University of California San Francisco, San Francisco, CA USA; 9 Division of Prevention Sciences, Department of Medicine, Center for AIDS Prevention Studies, University of California San Francisco, San Francisco, CA USA

**Keywords:** Perinatal depression, Mental health, Longitudinal qualitative, Financial insecurity, Food insecurity, Women living with HIV, Exclusive breast feeding, Prevention of mother to child transmission

## Abstract

Globally, depressive symptoms among pregnant and postpartum (i.e., perinatal) women living with HIV (WLWH) are alarmingly high and associated with poor outcomes such as suboptimal adherence to antiretroviral therapy (ART), and early cessation of exclusive breastfeeding (EBF). Few qualitative studies have described the experience of perinatal depression among WLWH to identify the underlying social-structural determinants of poor mental health and potential strategies to intervene. We conducted a longitudinal qualitative study applying semi-structured interviews with 30 WLWH at three timepoints (28–38 weeks pregnant, 6-weeks postpartum and 5–7 months postpartum) to understand mental health experiences of perinatal WLWH in western Kenya. Financial insecurity emerged as the central theme impacting the mental health of women across time. Financial insecurity was often attributed to the loss of employment, related to pregnancy and the demands of breastfeeding and caring for an infant, as well as a lack of support from male partners. The loss of income and subsequent financial strain contributed to worsening levels of food insecurity and relationship stress and challenged engagement in HIV care. In this way, increased financial strain during the perinatal period negatively impacted the mental health of perinatal WLWH. Our findings suggest support to meet basic needs and remain engaged in HIV care during pregnancy and postpartum could improve perinatal mental health for WLWH in this setting.

## Introduction

Poor mental health, including depression, is a pressing global health issue among pregnant and postpartum (i.e., perinatal) women living with HIV (WLWH) [1–3], particularly in sub-Saharan Africa (SSA) where the prevalence of HIV among women is disproportionately high [4]. Currently, 36% and 21% of WLWH experience depressive symptoms during pregnancy and the postpartum period, respectively [1]. Depression among perinatal WLWH has substantial implications for maternal and child health [5], as depression is associated with HIV disease progression [6, 7] as well as non-adherence to antiretroviral therapy (ART) [8, 9], and reduced exclusive breastfeeding (EBF) [10, 11], critical components of the prevention of mother to child transmission of HIV (PMTCT) strategies [12, 13].

In SSA, unplanned pregnancies [14], marital status [14, 15], difficulties with primary partners [16, 17] and social-structural factors such as food insecurity [17–19] and low educational level [20] have been associated with poor mental health among perinatal women. Yet, little is known about the lived experience of perinatal depression and HIV, how maternal mental health may vary across the perinatal period, and how social-structural factors such as food insecurity intersect with mental health and HIV to impact maternal and child health, including adherence to lifesaving ART. This knowledge is key to addressing the mental health needs of WLWH at different stages of the perinatal period in settings like SSA where social-structural factors have been shown to affect HIV-related health outcomes and mental health [21, 22]. To fill this gap and inform future interventions, we conducted a longitudinal qualitative study with perinatal WLWH in western Kenya exploring the mental health trajectories from pregnancy across the postpartum period.

## Methods

### Setting

This study was conducted at a subcounty hospital in Kisumu, Kenya in the former Nyanza region, where both HIV and poverty are widespread public health concerns. It is estimated that 17.4% of women in Kisumu county are living with HIV [23] and more than 40% of households report not having sufficient quantity of food or money to procure food [24]. We purposively recruited thirty women from Lumumba subcounty hospital in Kisumu who were at least 18 years old, between 28 and 38 weeks pregnant, living with HIV and with varying degrees of prenatal depressive symptoms to explore whether and how variations in prenatal depressive symptoms and other mental health symptoms such as stress and anxiety changed across time.

### Data Collection

Between March 2019-March 2020, we conducted semi-structured interviews lasting between 60 and 90 min at three consequential time points (i.e., 28–38 weeks pregnant, 6-weeks postpartum, and 5–7 months postpartum). The interviews elicited information on a range of topics based on our previous research [25, 26], and review of the literature. The interview guide included questions pertaining to the women’s feelings about their pregnancy and their HIV status, their experiences with HIV care, social support and access to food. A female research assistant fluent in Dholuo and KiSwahili conducted the interviews, which were audio-recorded, transcribed, and translated into English. Depressive symptoms were assessed at each time point using the validated Patient Health Questionnaire-9 (PHQ-9) instrument [27].

### Data Analysis

Interviews were coded using the Dedoose [28] software program based on an a priori coding schema while also allowing for emergent codes. The a priori codes were generated based on our interview guide and new codes emerged during our in-depth reading of the transcripts as we identified concepts, ideas and experiences that appeared frequently in the women’s responses. First, a thematic analysis [29] of the baseline data was conducted to identify emerging themes and to inform the 2nd and 3rd interviews. Thematic analyses were then completed using data from each subsequent interview. Through these cross-sectional thematic analyses, the central theme and its sub-themes from each timepoint were established. Using the framework analysis approach [30, 31], we then conducted a longitudinal analysis investigating changes in the central theme across time within individuals and among the group. A data matrix was created for each participant where sub-themes were rows and the timepoints were columns (see Table 1 for an example). Using the data matrices established for the 30 women, the research team worked collaboratively to describe the range of experiences within each sub-theme at each timepoint and then describe changes or consistencies in the sub-themes across timepoints. Specifically, we aimed to describe the experience portrayed by the majority of women and how and why the experiences changed or did not change across time. We also considered the contributions of exceptional cases in terms of how and why they differed from the experiences of the majority. The outcome was a detailed description of the central theme’s impact across time on the mental health experiences of pregnant and postpartum WLWH.

**Table 1:** Example individual participant summary table using framework analysis*

	28–38 weeks pregnant	6 weeks postpartum	5–7 months postpartum
PHQ-9 scores	14 (moderate depression)	6 (mild depression)	5 (mild depression)
Sub-themes of financial insecurity			
Disruption of income	She is not working as she gave up work when she started feeling sick with her pregnancy. She has limited access to preferred foods together with challenges accessing the HIV clinic. She plans to go back to work soon after delivery	She is employed in the informal sector, mostly doing laundry, and takes her baby and babysitter along. She now has better access to food and HIV care *‘P:… I have a job and I can eat what I was not able to before.’*	She continues working, now cooking and selling food. She has access to adequate food. Though, reports struggling to pay the rent and balance work and infant care
Unplanned pregnancy	She has an unplanned pregnancy (occurred during the first month after long term contraceptive was implanted). She feels this destroyed her life by interrupting her work. She has poor sleep and is worried about giving birth		
Worries about PMTCT	She was recently diagnosed with HIV, and her partner left when she disclosed her status, he says he does not know his own status and blames her. Her daughter picks up her medications at times when she lacks fare. There was also a day that once of the nurses had to pay for her fare *‘P: I am having problems with my husband because of my status which I told him and now that I got pregnant, we are having more problems, I mean he is not supporting me because he thinks I deliberately brought about the disease.’* She feels weak when taking her medications without food She is worried about how she will EBF when she needs to work and how this will affect the baby’s health and HIV status. She has never EBF before. She is worried about the transmission of HIV to her baby and this is her first pregnancy while living with HIV	She has better access to her HIV care having resumed work. She struggles to balance work with EBF, understanding EBF is important for preventing HIV transmission to her baby She feels she has milk insufficiency and also wants to supplement so she can work more. EBF interferes with doing things and she faces pressure from her daughter to supplement. Though she fears HIV transmission to her baby. She seeks the healthcare provider’s advice about the traditional practice of having her baby’s plastic teeth removed	Her partner later accepted her status, but he never got tested or never disclosed his status to her if he did. She experiences HIV and BF related stigma at work but her knowledge about HIV helps her to overcome *P: It was hard because sometimes I would get customers who feel bad when they see you breastfeeding the baby and serving them, some would not eat because they feel that you have touched your breasts then you are serving them and there were those who would just tell you to serve them first then you breastfeed the baby.’* *‘P: It is painful, but you just have to do what the customer demands because if you do not you will not get what to eat so that the baby gets milk.’* She started supplemental feeds from 3-months because of her feelings of insufficient milk. Her baby’s 1st test is HIV negative and she is not worried anymore. She had baby’s plastic teeth removed against the provides advice
Inadequate support	Married, then separated during her pregnancy. She feels her partner left when he found she was HIV + . This was their first child together. Her partner’s support is minimal to, at times, none. Her mother and daughter provider better support	She and her partner are not in communication, neither is he providing support. She texted him to inform him she gave birth and he did not respond. Her daughter and landlady/ friend provide good support	She is receiving support from her mother and daughter. Her partner came back for a few months, supported her, then left with no continued support

*Details limited and direct quotes removed to provide an analytical example while maintaining participant confidentiality

## Results

**Fig. 1:**
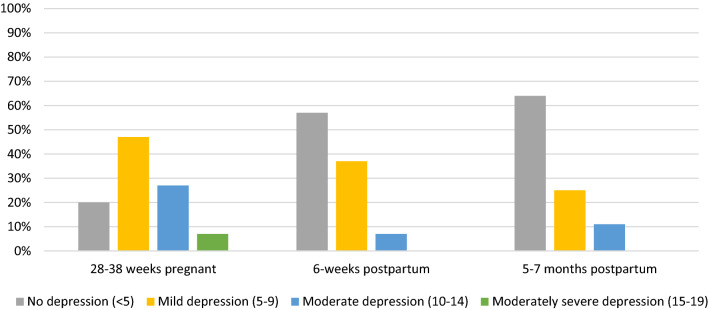
Depressive symptom scores using the patient health questionnaire 9 (PHQ-9)

Thirty women participated in 88 interviews over three time points spanning from the third trimester to 5–7 months postpartum (two participants moved and could not be reached for their final interview, data from their first two interviews were included in analyses). Of the 30 women, seven were first-time mothers and nine had received a diagnosis of HIV during the current pregnancy (see Table 2 for participant characteristics). At the first interview, 80% of the women reported symptoms indicative of probable depression. The prevalence of probable depression decreased to 43% at 6-weeks and 36% at 5–7 months postpartum (see Fig. 1).

**Table 2:** Sociodemographic characteristics of participants (n = 30)

	Before pregnancy	Pregnancy	6 weeks	5–7 months
		(n = 30)	(n = 30)	(n = 28)
Employment				
Employed	25 (83%)	6 (20%)	4 (13%)	12 (43%)
Unemployed	5 (17%)	24 (80%)	26 (87%)	16 (57%)
Employment type				
None/housewife	5 (17%)			
Office work	3 (10%)			
Trader	13 (43%)			
Day laborer	9 (30%)			
Ethnic group				
Luo		29 (97%)		
Kisii		1 (3%)		
Education				
None		17 (57%)		
Primary		5 (17%)		
Secondary		5 (17%)		
College		3 (9%)		
Unknown				
Time engaged in HIV care				
≤ 2 years		9 (30%)		
> 2 years		21 (70%)		
Children living at home				
None		7 (23%)		
1–3		18 (60%)		
> 3		5 (17%)		
Relationship status				
Living with partner		17 (57%)*	16 (53%)	14 (50%)
Not living with partner		13 (43%)	14 (47%)	14 (50%)

			M	SD
Age (range 18–37)			28.8	
Prenatal depressive symptoms (PHQ-9)			8.2	4.0
6-weeks depressive symptoms (PHQ-9)			4.8	2.7
5–7 months depressive symptoms (PHQ-9)			4.1	3.2

*Four women reported polygamous relationships at pregnancy timepoint

### Central Theme Across Time: Financial Insecurity

Financial insecurity emerged as the central theme impacting the mental health experience of WLWH across the perinatal period. Financial insecurity was comprised of several sub-themes at each time point including, Disruption of income, Unplanned pregnancy, Worries about PMTCT and Inadequate support. These sub-themes progressed across time as women’s transition through the perinatal period evolved (see Table 3).

**Table 3:** Central theme with progression of subthemes across the perinatal period

Central theme across time: financial insecurity			
	28–38 weeks pregnant	6-weeks postpartum	5–7 months postpartum
Sub-themes with description across time			
Disruption of income	Loss of employment related to pregnancy leads to increased food and financial insecurity for the family	Unemployment and food insecurity persist due to continued lack of income	Hopeful about resuming work despite challenges, yet food insecurity persists for most
Unplanned pregnancy	The impact of unplanned pregnancy		
Worries about PMTCT	Financial and food insecurity increase worries about PMTCT	Financial and food insecurity persist, sustaining worries about PMTCT	Worries about PMTCT are reduced but still present
Inadequate support	Abandonment and inadequate support	Support insufficient to compensate for lost income, difficult to rely on others	Continued difficulties relying on others

### Financial Insecurity in the Third Trimester

#### Disruption of Income: Pregnancy Related Unemployment Increases Food and Financial Insecurity

The majority of women (83%) were employed prior to pregnancy. However, by the 28th–38th week of pregnancy, most employed women (76%) had left the workforce, primarily because of physical limitations related to their pregnancy. Their departure from the workplace led to a disruption of income, which created stress, anxiety and worry because women and their dependent children relied on this income for basic needs including, food, rent, transportation to antenatal and HIV care appointments, primary school fees, and water. Most women had at least one young child at home whereby the loss of income caused worry not only about their own very basic needs, but also those of their children as expressed by participant 025,

“I am fatigued, I feel stressed and I am also experiencing many challenges like accessing food… what is mainly stressing me is not having a source of income and my baby’s father abandoned me. When I came to the hospital, I was told that my hemoglobin level was low so I am worried about what I will do after giving birth or even how my children will survive”.

Many women described sadness, frustration and embarrassment over not being able to provide food and school fees for their children (see Table 4 for additional illustrative participant quotes). The loss of income led to both physical and mental anguish for women during their pregnancies and many women reported plans to rapidly return to the workforce after giving birth as a means of improving their access to food and ability to meet the needs of their children as seen in participant 08,

**Table 4:** Quotes from the qualitative semi-structured interviews illustrating the central theme: financial insecurity “When you have children, you will have to think about how they can survive or how they can access food and there is no money, when they are still young then they will even cry, so you will be stressed about how to access food with no money.”

28–38 weeks pregnancy	6 weeks postpartum	5–7 months postpartum
**Disruption of income: pregnancy related unemployment increases food and financial insecurity** *19: “P: I would get fatigued and dizzy, that work is very hard and because I was always dizzy, I had to stop. I was considering getting a job when the baby is three months old so that I am able to sustain the baby.”* *24: “P: Yes, that happens like for me, I used to do business, but I stopped so you have to be depressed. You get depressed because it is something that you go back to after giving birth.”* *25: “P: I am praying to God so that I have a safe delivery because my plan is to start a business, I had gotten a stall that I would start my business in after my baby turns one week old, provided his/her umbilical cord had detached from his/her navel. I had said that if I had only these children and I opened a business in a stall then I would grow.”* **Unplanned pregnancy** *25: “P: I got pregnant while on implant. I used to see myself growing fat little did I know that I had conceived already. I could not fit into my clothes and at 5 months, I started feeling something moving around in my womb. I decided to go to a chemist in carwash (name of place) to buy a pregnancy test kit and after testing, I found out that I was pregnant. From that time, I started feeling stressed and my partner had already abandoned me.”* **Worries about PMTCT: increased by food and financial insecurity** *04: “P: You know at times, let me say you want to go and get your medication from the clinic and you do not have money, at times you have to get someone who can support you or give you the money, I mean the means of transport to get to the health center.”*	**Disruption of income: Unemployment and food insecurity persist** *09: “P: I plan to go back, when the child is weaned and I get some money, I will go back.”* *01: “P: I will try to look for a job closer to me so that the person whom I shall leave with the baby can come over but not now, for now, I want to be with her for six months until she starts eating, then it will be better.”* *28: “P: Being without a job is difficult like right now it’s very difficult because of the baby. When I didn’t have the baby, I told you that I could go and look for a casual job, sometimes I could wash clothes, so you know right now it is not possible.”* *09: “P: Yes, previously I would go sell and we would have some extra cash to spend; when my husband did not have I would supplement but now, he is the only one working and sometimes there is no job so it is tough and I have to worry about that.”* *09: “P: I only worry when my child is out of school because of school fees and my neighbor’s children are going to school, this makes me sad and I worry a lot.”* *016: “‘P: As a woman there are things that you desire to have but because you have financial challenges you are not able to buy the things that you want; for example, you may not be able to buy enough food for your children and sometimes you have seen something good in the market that you want to have but you cannot buy it because you lack money.’* *22: “‘P: I am worried because sometimes I feel hungry but there is no food to eat.’* **Worries about PMTCT: food and financial insecurity persist** 01: “P: It has happened, like the last time I came, you really helped me, I can remember I did not have money for transport, I had walked from the road [FO: Pointing towards a direction] until here. You helped me with means back home.” *03: “P: What is troubling me is having a source of income but if I can get a good source of income then I can be okay so when my appointment day comes, I will have money for transport to go to the hospital as scheduled, I don’t want to miss.”* *28: “P: I don’t get fare easily; I try and if I don’t get money to board a motorbike then I look for 20 Kenyan shillings (KSh) for boarding a vehicle…Once I get fare for coming, I walk when going back and that’s why I tag along with his sister because it’s easier as we carry the baby in turns.”* *25: “P: I am still feeling stressed because I am supposed to be breastfeeding him for six months and for him its exclusive and right now, he is 6 weeks and sometimes we sleep hungry. This bothers me that if I can get something [referring to a job] then I can just start working with him.”*	**Disruption of income: hope about resuming work despite challenges** *012: “P: Right now, I can go and look for something because the baby is big and he can take porridge so after I make something for him, I just leave him with someone and then I go look for work, which I could not do when I was pregnant but now I can.”* *017: “P: Right now, I have the thought of even starting a business just that my baby is still young* *06: “P: Let me say like right now I am just begging for help, begging is not good, if I can get some little capital, I can start a business and I can carry my baby with me and go with him to my work place, and I can do my work without giving people a burden. I feel bad that I should not be begging.”* *029: P: I would like to get more support but you cannot ask for it every day because you will be given maize or food and it will get finished, I would like them to give me capital so that I can start something for my own so that I do not have to depend on them, but right now I know it is not possible because they [referring to her husband’s second family] have three people in the university* *025: “P: Yes, right now it is not as before, I may not be able to pay the rent but we can get food, not as before when we would go hungry, right now we can get porridge and drink at times we would take black tea and maize and sometimes when the maize dries, we can take it to the flour mill then make ugali.”* *029: “P: We don’t have a problem with food, I farm and when I have maize I am not worried, because when there is flour in the house then you can take one chicken that you have and sell, sometimes you can sell the vegetables and you will have some ease so when you farm you can sell some of the produce you have and be able to buy 'blueband'[Margarine] and whatever else that you need.”* *030: “P: I have been worried about that because I do not know where I am going to get the food for the baby because I am not even able to get food that I can eat so where would I get the one for the baby, I even joked with the doctor that should I cut my thigh so that I can give the baby. I really do not know where I am going to get the food, if I had money then I would have gone and bought flour for the baby. Where am I going to get flour and milk?* *011: “P: Yes, I am having a challenge because you have to eat well in order to have enough breastmilk and the baby breastfeeds a lot, after a few minutes, he pisses and then it is fully utilized in his body then he has to breastfeed again so this is a big challenge for me to exclusively breastfeed because the baby may want to breastfeed and I don’t have milk* **Worries about PMTCT: reduced, but present** *020: “P: Sometime I am supposed to come to the clinic and I do not have money for fare so I do talk to a ‘boda boda’[motorcycle taxi] man, he is my customer, so I talked to him so he brings me up to here.”*
	**Inadequate support: difficult to rely on others**	
	*26: “P: I have had so many challenges, my husband left me and sometimes he can only send me 400 KSh in a week and sometimes he does not send; I decided not to be sad about it and not to depend on him I have to look for a way to be independent.”*	
26: “P: Sometimes I don’t have money for fare that’s why I send my daughter to come and collect my medicines for me because for me to walk to ‘Riat’ [Name of place] it will take time or I will be late. If I don’t have fare, I tell myself that I will come the following week but I do receive a phone call asking why I did not go to the go to the clinic for my medication. They will tell you that you are given one month to plan on how to get fare but sometimes you were expecting some money but you don’t get it and that is why I send my daughter because I know she can walk from the stage to this place then come back	*19: “‘P: When my sister has no money, the food will be less in the house.’ I: Have you ever experienced hunger on a daily basis?” P: It can be twice in a week.”*	*17: “P: There was a day I lacked fare and missed to come but I came the following day.”* **Inadequate support: continued difficulties relying on others**
25: “P: What can make it hard is if I get a job because I will have to carry the baby to work so that he/she does not have a hard time because the baby will be breastfeeding. Another thing is that I must have breastmilk constantly though sometimes I may not have food to eat and I need food to have enough breastmilk because the baby will be breastfeeding exclusively without giving even water for 6 months.”	*16: “P: I need support with how to feed the baby and basic needs in the house because her father goes to work but sometimes, he does not get enough.”*	*019: “P: Like support with the baby. If I ask her for money to buy something, she doesn’t give me….sometimes she says she doesn’t have.”*
**Inadequate support and abandonment**	*20: “‘P: Yes, sometimes I am not given and sometimes if you are given a lot then its 100 KSh so its upon you to see how you will use the 100 shillings. Once you are given the 100 shillings, it will take a week before you are given another one.’*	*22: “P: It is not easy because we are depending on one person to bring but at times you can wait for him to bring but he does not.”*
025: “P: Sometimes it gets tough because sometimes I am hungry and ask hoping that I will get but they tell me they still do not have money and that I should wait until he/she gets. Sometimes it takes even one week before he/she sends you money so if you feel like eating something, you can take it on credit from the shop but it becomes difficult to go to the shop because you have too much debts for the shop.”	*17: “P: It was challenging because waiting for the money is not easy, waiting for him until he gets back, unsure whether he is going to come back with some money or he will not make any, it is a challenge.”*	*021: “P: Okay, right now I feel bad because I feel like I’m giving my parents a burden now. I can remember going to school, you are being provided for then after that, maybe they had hope that after you finish and you would get some jobs. Then, all of the sudden, you are still there. You have added another member of the family and all these things they are seeing now, like as for me, I feel like a burden to them.”*
24: “P: I must have concerns because I was used to having my own income and I could eat whatever I felt like. For example, if you are working, you can buy anything let’s say you are feeling like eating meat or fish you will easily buy but if it’s the man providing, he may leave for you money to buy vegetables even if you didn’t feel like it.”	*04: “P: It is difficult to ask for money because when you need to do something, you will have to ask for money and maybe the person you are asking may not be having money at that time so it is really difficult but if you had your own money then you would take care of your needs on your own.”*	*015: “P: You just feel like everything is good when you have your money and you are not depending on a man’s money.”*
28: “P: After the baby has grown, I will go back to work and this would boost us, you know it is a burden just depending on one source of income and sometimes it is not even enough.”	*01: “P: I don’t feel good because I used to be an independent woman, asking for money is difficult.”*	*13: P: My parents support me in everything now that I am not working, my mum buys food for the baby, she buys him clothes and she feeds me*

#### Unplanned Pregnancy

There was an added layer of hopelessness, stress and sadness for most women whose pregnancies, and therefore reductions in household income, were unplanned. Unplanned pregnancies were especially difficult to accept for nearly a third of the women who conceived while using long-acting contraception. Several women also reported being abandoned by their partners after revealing their unplanned pregnancy as expressed by participant 025,

“I got pregnant while on implant. I used to see myself growing fat little, but I didn’t know that I had conceived already… I decided to go to a chemist in carwash (name of place) to buy a pregnancy test kit and after testing, I found out that I was pregnant. From that time, I started feeling stressed and my partner had already abandoned me”.

Women described mental health symptoms such as feeling sad and stressed about their untimely pregnancy, its impact on their ability to earn an income, or the anticipated burden of providing for the child. At least 7 women experienced severe financial strains and food insecurity which led them to deliberate over the difficult decision of whether or not to terminate the pregnancy.

#### Worries About PMTCT: Increased by Food and Financial Insecurity

Women also described how financial insecurity caused stress and anxiety by making adherence to ART difficult. Women were highly committed to their HIV care and reported that worry about vertical HIV transmission was a primary motivator for their own adherence. Yet, many women described being unable to pay for transportation to the clinic and struggling to walk to the clinic during late pregnancy. In addition, without money for food, women experienced dizziness, nausea and other uncomfortable side effects from taking ARTs while hungry. While struggling to remain adherent to ART, women also worried about how their lack of money and food would impact their ability to protect their babies from HIV by EBF for the first six months. Most women were uncertain if they would have enough food to ensure adequate breastmilk supply and many feared that EBF would interfere with their return to work as heard by this mother (025),

“Another thing is that I must have breastmilk constantly though sometimes I may not have food to eat and I need food to have enough breastmilk because the baby will be breastfeeding exclusively without giving even water for 6 months.”

#### Inadequate Support and Abandonment

During this period of financial insecurity, when women might turn to their partners for support, there were only five women who were in committed partnerships where the wellbeing of the family was a shared priority *and* their partners were able to provide enough support to meet the family’s basic needs. The remaining women described partners who were unwilling or unable to compensate for their lost income, with many women expressing uncertainties about how much their partner was earning or how he was allocating his income. Some women reported partners having extra-marital affairs or having drinking problems, and believed their partner was diverting his funds to these activities rather than providing for their family. Nearly half of the women were not living together with the father of their baby. This was either because they were separated, they were a secondary wife in a polygamous marriage, they had yet to move in together, or they had been abandoned completely. For a third of the women in our cohort, their partner abandoned them after discovering they were pregnant as seen by this example (019),

“I went to the hospital, got tested and I was told that I was pregnant. I felt bad since I was not ready to have a child and [when I told the father] he told me that he was not ready to be a father- that is the reason we broke up”.

For women who were abandoned as such, this also marked their first pregnancy with the father of the baby. Women attributed being abandoned to a variety of reasons including, the partner not being ready for or wanting a child, disclosing their HIV status to their partner, or the increased pressure on partners to provide financial support after women stopped working during pregnancy.

Without support from the father of the baby, women were forced to turn to others such as neighbors, friends, relatives or in-laws for food or money and they were met with varying degrees of support. Many women felt uncomfortable asking others for help and became increasingly anxious by their accumulating debts. Notably, some women were received poorly by family when they sought needed support. Meanwhile, others were reluctant to ask for help fearing their family’s reactions to their pregnancy and need for support. Most women, even those receiving adequate support, still desired the security of having their own source of income over the uncertainty and discomfort of relying on others.

### Financial Insecurity at 6-Weeks Postpartum

#### Disruption of Income: Unemployment and Food Insecurity Persist

At 6 weeks postpartum, financial insecurity persisted or worsened for the majority of women. Most women remained unemployed often citing the need to EBF as a primary reason for delaying their return to work as expressed by this mother (001),

“I will try to look for a job closer to me so that the person whom I shall leave with the baby can come over but not now, for now, I want to be with her for six months until she starts eating, then it will be better”.

Two-thirds of women reported struggling to access food due to a lack of income and half explicitly said their family missed meals or even went full days without food. Women expressed continued feelings of worry about their other dependent children and having adequate amounts of money and food to meet the family’s basic needs. Of the four women who returned to work, three were unable to earn enough to notably reduce their financial and food insecurity. Only one woman was able to return to work and change her situation such that she reported less stress from financial insecurity. This woman also received substantial financial support from friends and family and was able to resume work as a housekeeper where she could bring her baby and a babysitter along to help her. Despite a solid support system, she reported struggling to balance work with EBF during this early postpartum time, eventually introducing supplemental feeds at 3-months postpartum. Although most women remained unemployed during this early postpartum period, nearly all expressed a desire or pressure to quickly return to work to provide financial support for their family.

#### Worries About PMTCT: Financial and Food Insecurity Persist

At this time, financial insecurity continued to cause women anxiety, stress, worry and feelings of hopelessness by challenging their HIV care and their adherence to the PMTCT guidelines. Although women remained engaged in HIV care, they continued to worry about the transmission of HIV to their infants. Furthermore, many reported continued struggles to pay for transportation to the clinic especially since infant care appointments were now separate from their own as expressed by this mother (028),

“I don’t get fare easily; I try and if I don’t get money to board a motorbike then I look for 20 shillings [U.S 20 cents] for boarding a vehicle…Once I get fare for coming, I walk home and that’s why I tag along with his sister because we carry the baby in turns”.

A lack of food to take medications with, continued to threaten adherence to ARVs, meanwhile prenatal worries about breastfeeding had developed into active struggles with EBF. Nearly half of the women reported they had milk insufficiency related to their own inadequate food intake and a third of women described fearing that their babies were not gaining weight and were frequently crying from what seemed to be hunger as heard by these women,

“I asked and I was told that there was nothing else I could give her, but sometimes I experience a challenge because the baby breastfeeds and night comes before I have even eaten anything since morning. I sometimes have pity on the baby because there is no way she can even add weight (025)”,

“[when my] baby is breastfeeding, and the breastmilk is not sufficient, he breastfeeds and cries because he does not get enough milk (022)”.

Some women wanted to introduce supplemental feeds because of milk insufficiency and many others faced pressure from family members to do so, creating feelings of fear, confusion, anxiety and worry as women understood EBF to be an important part of PMTCT. EBF was also in jeopardy for women who considered returning to work to improve their access to food.

#### Inadequate Support: Difficult to Rely on Others

Meanwhile, most women continued to report that their partners, regardless of relationship status (e.g., living together, separated, or polygamous), provided insufficient support for meeting their basic needs. Several women reported receiving less support than during pregnancy or noted a deterioration of their relationship or worsening emotional or verbal abuse. Thus, for the majority, partners provided little relief from the physical and psychological burden of severe financial and food insecurity. Even among the few who reported receiving support from their family, there continued to be an underlying discomfort, stress, or feeling of insecurity that came with relying on others as reflected by this mother (017),

“It was challenging because waiting for the money is not easy, waiting for him until he gets back, unsure whether he is going to come back with some money or he will not make any, it is a challenge.”

### Financial Insecurity at 5–7 Months Postpartum

#### Disruption of Income: Hopeful About Resuming Work Despite Challenges

For many women, 5–7 months postpartum seemed to be a turning point. The negative impact of financial insecurity on mental health, including depressive symptoms, had reduced for nearly a third of the women (see Table 2 / Fig. 1 for PHQ-9 results) and most described new hopefulness and relief that the prescribed EBF period was ending and they could look for work or resume work at full capacity. Only four women, who were married and living together with their partners, described plans to further delay their return to work in order to care for their baby. The remaining women were imminently planning to resume work despite the many challenges faced in rejoining the workforce. For nearly half of the women, a lack of start-up capital was a major barrier to resuming work. Women, who had been buying and selling goods such as food or clothing prior to pregnancy, were ready to restart their small businesses. However, many explained that during the prenatal and early postpartum period, when they were unable to work, they used the capital reserved for their businesses for food and other expenses (see Box 1). Women also described challenges balancing work with infant care and some reported they had returned to work bringing their babies along. Despite difficulties, more than a third of the women had resumed work in some capacity. Yet, less than half of those who returned to work reported markedly improved household income or access to food. Most of the women who had returned to work still reported ongoing worries about finances as they were underemployed and still struggling to make ends meet while hoping for a better job or additional work.

**Figure:**
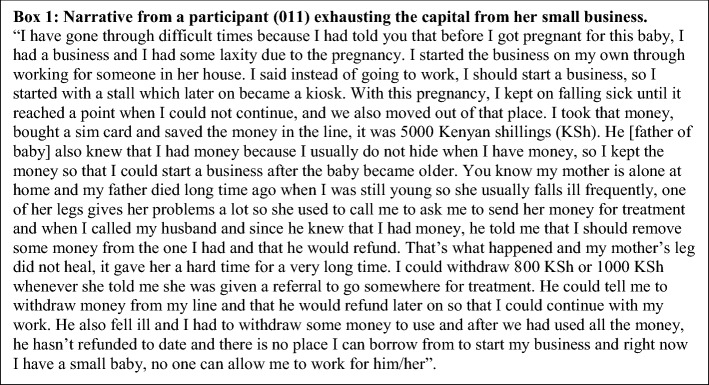

#### Worries About PMTCT: Reduced, but Present

Despite hopefulness, and for some the return to work, the majority of women reported that financial insecurity continued to challenge their HIV care, though to a lesser extent than during pregnancy or early postpartum. Although most women still described difficulties accessing fare to the clinic, walking was now easier than during pregnancy or just after birth. Also, fewer women described difficulties taking ARTs when hungry. Worries about HIV transmission to their infants had also decreased by this time, as the results of the baby’s first HIV test had been confirmed negative for the 28 women whom we interviewed at this timepoint (two participants were lost to follow up). Additionally, despite ongoing worries about not having enough food to produce adequate breastmilk, the majority of women persevered and were able to EBF for the first six months, with most women expressing relief that they had reached the end of the recommended EBF period. However, with the end of EBF, especially for those who were most financially insecure, new worries emerged about how to access cow’s milk, formula or other supplements for the baby.

#### Inadequate Support: Continued Difficulties Relying on Others

For some women, a lack of support from the father of the baby persisted and continued to cause sadness or stress. For a few women their partners or family started to provide some support helping to alleviate stress, including several women who relocated to live with family members who could better support them. For a few other women, their partner’s income increased, or the relationship had improved which led to better financial support. Regardless of the level of support they were receiving, women continued to feel financially insecure without their own source of income—often citing either insufficient support, unreliable support or other negative feelings that came with depending on others such as embarrassment or being a “burden”—simply put by participant 015,

“You just feel like everything is good when you have your money and you are not depending on a man’s money”.

## Discussion

Our objective was to understand the mental health experiences of WLWH in western Kenya across the perinatal time period. We found increased financial insecurity attributed to the pregnancy and postpartum period was the most prominent theme impacting mental health among WLWH. Moreover, our findings demonstrate women’s departure from the workforce (and resultant loss of income) during late pregnancy triggered acute stress, sadness and depressive symptoms as women were no longer able to meet their families’ most basic needs. These negative feelings persisted into the immediate postpartum period (6-weeks), then generally reduced as women made plans to resume or had resumed work. By 5–7 months postpartum, women expressed relief that they’d completed the 6-month EBF recommendation bringing about newfound hope that they had protected their babies from contracting HIV and returning to work was in sight. Across the perinatal time frame, feelings of hopelessness, worry and sadness from the strain of financial insecurity were intensified by factors such as having dependent children to provide for, unplanned pregnancy, a lack of support from the father of the baby or other family as well as living with HIV, which for nearly a third of women was a new diagnosis.

**Fig. 2:**
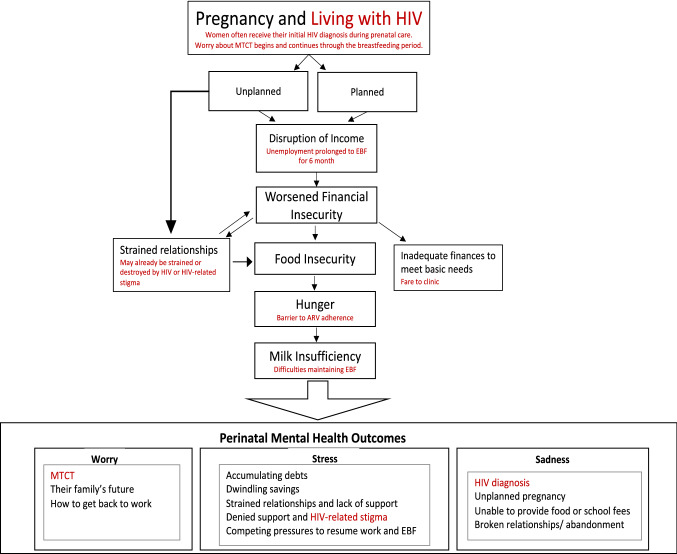
Conceptual pathway of perinatal mental health among WLWH

Our study offers a novel finding that identifies women’s departure from the workforce during pregnancy as a primary factor impacting symptoms of perinatal mental health. Although more than half of our sample of women had only a primary school education or less (limiting employability and access to higher paying jobs) and more than two-thirds worked strictly in the informal sector, their income (however small it may have been) was critical to their family’s survival prior to pregnancy. Studies have linked unintended pregnancy [32], lack of support [33], financial constraints [32] and food insecurity [34] to poor perinatal mental health for WLWH in similar settings. However, we have exposed a pathway (see Fig. 2), clearly described by women, whereby pregnancy interrupts gainful employment, which in the setting of inadequate support, increases financial insecurity leading to stress, hopelessness and worry about paying for food and other basic needs to support their infant and other dependent children. The negative impact of the loss of employment on women’s mental health may also be a reflection of women’s feelings of empowerment from personal income and the subsequent feelings of disempowerment that may arise from the leaving the workforce.

Furthermore, our findings reveal that stress and worry from financial insecurity were acutely worse when the pregnancy and subsequent income loss was unplanned. This was especially true when unplanned pregnancy was coupled with being abandoned by the father of the baby (as was the case for a third of the women). Moreover, the majority of women who experienced unplanned pregnancy and abandonment were also simultaneously coping with a new diagnosis of HIV. Qualitative descriptions indicated that unplanned pregnancy, abandonment and a new HIV diagnosis caused women the greatest stress and sadness during pregnancy with the impact of all three fading across time. Meaning, with time, women came to accept their HIV diagnosis, view their pregnancy more positively and come to terms with being a single mother after being abandoned by the father of their baby. Indeed, several other studies have linked unintended pregnancy to antenatal depression [35] and this may explain why our PHQ-9 scores show more women were experiencing symptoms indicative of depression during pregnancy than postpartum. Importantly, PHQ-9 scores dropped substantially across time despite women reporting continued worry and stress related to financial and food insecurity at 6 weeks and 5–7 months postpartum. This supports the idea that the negative impact of circumstances during pregnancy on women’s mental health (i.e. unplanned pregnancy, abandonment or a new HIV diagnosis) reduced across time and/or women developed coping strategies to mitigate their depressive symptoms. We also found women’s need for financial and food support to compensate for lost income, was primarily unrelieved by partners or family whose support was insufficient, unreliable or too shameful to solicit, thus contributing to a sense of being alone and loss of security previously felt from personal income. This highlights the need to better understand women’s relationships with the babies’ fathers during the perinatal period including the social and cultural context, especially in this setting where polygamy is an acceptable practice and where single motherhood [36, 37] is both highly prevalent and associated with poor outcomes including mental health.

Our study expands on current evidence to lend important insight into how stress and sadness related to financial insecurity uniquely impact WLWH during the perinatal period and how it unfolds across time. We show that the mental health impacts from worsening financial and food insecurity are magnified for WLWH as a lack of money and food made it increasingly difficult for women to adhere to the PMTCT guidelines, thereby increasing their worries about MTCT [3, 32]. Additionally, women were under pressure to adhere to the recommendation from providers to EBF for 6-months (a public health and PMTCT recommendation), yet, this became progressively challenging across time as the period of financial and food deficits persisted. Women faced a dilemma: returning to work would interfere with EBF, but without income from work they felt their access to food was inadequate to support sufficient breastmilk production. This was further complicated by the fact that the women received little to no instruction from healthcare providers about how to practice EBF, alternative infant feeding options, or even general breast health or breastfeeding practices. In addition, evidence shows an inadequate support system for middle-income and low-wage working mothers in Kenya who wish to continue breastfeeding while returning to work [38, 39]. Thus, women who prioritized EBF over work faced prolonged unemployment and corresponding food insecurity. This is in line with findings from Garcia et al. [34] who showed increasing household food insecurity among postpartum mothers living with HIV. We go beyond these findings to show a link between food insecurity and the stress and anxiety of perceived milk insufficiency during the EBF period which persist to 6-month postpartum.

### Public Health and Policy Implications

Our study highlights the importance of access to safe and reliable family planning. Despite improvements in access to family planning in Kenya [40], two-thirds of women in our cohort had unplanned pregnancies. In addition, nearly a third of women reported using long-acting contraception at the time they became pregnant (most often monthly injections or 3–5-year implants). Importantly, ongoing research is investigating the potential effect of ARTs on different types of contraception [40–42]. This is urgently needed as seen here by the number of pregnancies occurring while on contraception (around 30%). Consequently, the substantial negative impact of unintended pregnancies on women’s mental health may have largely contributed to women’s symptoms indicative of probable depression being twice as prevalent during pregnancy than postpartum (see Fig. 1). In addition, male partner involvement and support was limited to nonexistent, especially among WLWH whose pregnancies were unplanned, placing them in further financial hardship during an especially vulnerable period of time. Above all, these findings illustrate the need for improved family planning support for women of childbearing age, including updated and targeted family planning options for WLWH.

Finally, our research indicates three areas where interventions and policies could support the perinatal mental health of WLWH: (1) financial and food support during late pregnancy and early postpartum, (2) interventions and policies to support EBF including more support and information from healthcare providers and support for working mothers and (3) access to capital and training for postpartum women re-entering the workforce as small-scale entrepreneurs including as vendors or through farming [43].

### Limitations

This study aimed to understand how the experience of pregnancy and the postpartum period impacts the mental health of perinatal WLWH. While our findings suggest that the 5th–7th month postpartum seemed to be a turning point for many, we were unable to follow-up with women at 9th–12th months as planned due to the limitations related to the COVID-19 pandemic. Nevertheless, we were able to capture and report an in-depth perspective of how financial insecurity, the most prominent theme, impacts WLWH’s mental health experiences during the perinatal period.

### Conclusions

Our study explored the mental health experience of perinatal WLWH across time using a longitudinal qualitative design. Financial insecurity was the prominent theme impacting the mental health, including depressive symptoms among our cohort of perinatal WLWH. Our findings show the loss of employment during pregnancy and subsequent financial strain is an important and overlooked factor impacting the food insecurity, HIV care, relationships and overall mental health of perinatal WLWH in low resources settings. Future research is needed to understand the relationship dynamics between perinatal WLWH and their partners as well as identify interventions that improve family planning services and support women to overcome financial hardship throughout the perinatal period.

## Data Availability

The qualitative data is not available for distribution.

## References

[1] Zhu QY, Huang DS, Lv JD, Guan P, Bai XH (2019). Prevalence of perinatal depression among HIV-positive women: a systematic review and meta-analysis. BMC Psychiatry.

[2] Sowa NA, Cholera R, Pence BW, Gaynes BN (2015). Perinatal depression in HIV-infected African women: a systematic review. J Clin Psychiatry.

[3] Kapetanovic S, Dass-Brailsford P, Nora D, Talisman N (2014). Mental health of HIV-seropositive women during pregnancy and postpartum period: a comprehensive literature review. AIDS Behav.

[4] Frank TD, Carter A, Jahagirdar D, Biehl MH, Douwes-Schultz D, Larson SL (2019). Global, regional, and national incidence, prevalence, and mortality of HIV, 1980–2017, and forecasts to 2030, for 195 countries and territories: a systematic analysis for the Global Burden of Diseases, Injuries, and Risk Factors Study 2017. Lancet HIV.

[5] Madlala SS, Kassier SM (2017). Antenatal and postpartum depression: effects on infant and young child health and feeding practices. S Afr J Clin Nutr.

[6] Rivera-Rivera Y, Vazquez-Santiago FJ, Albino E, Sanchez MD, Rivera-Amill V (2016). Impact of depression and inflammation on the progression of HIV disease. J Clin Cell Immunol.

[7] Antelman G, Kaaya S, Wei R, Mbwambo J, Msamanga GI, Fawzi WW, Smith Fawzi MC (2007). Depressive symptoms increase risk of HIV disease progression and mortality among women in Tanzania. J Acquir Immun Defic Syndr.

[8] Whetten K, Shirey K, Pence BW, Yao J, Thielman N, Whetten R (2013). Trauma history and depression predict incomplete adherence to antiretroviral therapies in a low income country. PLoS ONE.

[9] Uthman OA, Magidson JF, Safren SA, Nachega JB (2014). Depression and adherence to antiretroviral therapy in low-, middle- and high-income countries: a systematic review and meta-analysis. Curr HIV/AIDS Rep.

[10] Tuthill EL, Miller JD, Collins SM, Widen EM, Onono M, Young SL (2020). HIV infection, hunger, breastfeeding self-efficacy, and depressive symptoms are associated with exclusive breastfeeding to six months among women in western Kenya: a longitudinal observational study. Int Breastfeed J.

[11] Madeghe BA, Kimani VN, Vander Stoep A, Nicodimos S, Kumar M (2016). Postpartum depression and infant feeding practices in a low income urban settlement in Nairobi-Kenya. BMC Res Notes.

[12] World Health Organziation. Updates on HIV and infant feeding. 2016. Available at: https://www.who.int/maternal_child_adolescent/documents/hiv-infant-feeding-2016/en/

[13] World Health Organization. The use of antiretroviral drugs for treating and preventing HIV infection: recommendations for a public health approach. 2016. Available at: https://www.who.int/hiv/pub/arv/arv-2016/en/27466667

[14] Manikkam L, Burns JK (2012). Antenatal depression and its risk factors: an urban prevalence study in KwaZulu-Natal. S Afr Med J.

[15] Bernatsky S, Souza R, de Jong K (2007). Mental health in HIV-positive pregnant women: results from Angola. AIDS Care.

[16] Ramchandani PG, Richter LM, Stein A, Norris SA (2009). Predictors of postnatal depression in an urban South African cohort. J Affect Disord.

[17] Azale T, Fekadu A, Hanlon C (2018). Postpartum depressive symptoms in the context of high social adversity and reproductive health threats: a population-based study. Int J Ment Health Syst.

[18] Abrahams Z, Lund C, Field S, Honikman S (2018). Factors associated with household food insecurity and depression in pregnant South African women from a low socio-economic setting: a cross-sectional study. Soc Psychiatry Psychiatr Epidemiol.

[19] Garman EC, Schneider M, Lund C (2019). Perinatal depressive symptoms among low-income South African women at risk of depression: trajectories and predictors. BMC Pregnancy Childbirth.

[20] Peltzer K, Rodriguez VJ, Lee TK, Jones D (2018). Prevalence of prenatal and postpartum depression and associated factors among HIV-infected women in public primary care in rural South Africa: a longitudinal study. AIDS Care.

[21] Tsai AC, Bangsberg DR, Frongillo EA, Hunt PW, Muzoora C, Martin JN (2012). Food insecurity, depression and the modifying role of social support among people living with HIV/AIDS in rural Uganda. Soc Sci Med.

[22] Weiser SD, Palar K, Frongillo EA, Tsai AC, Kumbakumba E, Depee S (2014). Longitudinal assessment of associations between food insecurity, antiretroviral adherence and HIV treatment outcomes in rural Uganda. AIDS.

[23] Kenya Ministry of Health. HIV Estimates Report. October 2018. Available at: https://africaopendata.org/dataset/kenya-hiv-estimates-2018/resource/97ab3a85-71bd-4c49-b52c-6d2898d05181.

[24] Kenya National Bureau of Statistics. Kenya Demographic and Health Survey. 2014. Available at: https://www.dhsprogram.com/publications/publication-fr308-dhs-final-reports.cfm.

[25] Murnane PM, Miller JD, Tuthill EL, Collins SM, Neilands TB, Onono M (2020). Perinatal food insecurity and postpartum psychosocial stress are positively associated among Kenyan women of mixed HIV status. AIDS Behav.

[26] Tuthill EL, Maltby A, Conteh J, Sheira LA, Miller JD, Onono M (2020). Persistent food insecurity, but not hiv, is associated with depressive symptoms among perinatal women in Kenya: a longitudinal perspective. AIDS Behav.

[27] Monahan PO, Shacham E, Reece M, Kroenke K, Ong'or WO, Omollo O (2009). Validity/reliability of PHQ-9 and PHQ-2 depression scales among adults living with HIV/AIDS in western Kenya. J Gen Intern Med.

[28] Dedoose web application for managing, analyzing, and presenting qualitative and mixed method research data. 7.0.23, ed. Los Angeles, CA: SocioCultural Research Consultants, LLC; 2016.

[29] Braun V, Clarke V (2006). Using thematic analysis in psychology. Qual Res Psychol.

[30] Lewis J (2007). Analysing qualitative longitudinal research in evaluations. Soc Policy Soc.

[31] Tuthill EL, Maltby AE, DiClemente K, Pellowski JA (2020). Longitudinal qualitative methods in health behavior and nursing research: assumptions, design, analysis and lessons learned. Int J Qual Methods.

[32] Ashaba S, Kaida A, Coleman JN, Burns BF, Dunkley E, O'Neil K (2017). Psychosocial challenges facing women living with HIV during the perinatal period in rural Uganda. PLoS ONE.

[33] LeMasters K, Dussault J, Barrington C, Bengtson A, Gaynes B, Go V (2020). "Pain in my heart": understanding perinatal depression among women living with HIV in Malawi. PLoS ONE.

[34] Garcia J, Hromi-Fiedler A, Mazur R, Marquis G, Sellen D, Lartey A, Perez-Escamilla R (2013). Persistent household food insecurity, HIV, and maternal stress in Peri-Urban Ghana. BMC Public Health.

[35] Brittain K, Mellins CA, Remien RH, Phillips T, Zerbe A, Abrams EJ (2019). HIV-status disclosure and depression in the context of unintended pregnancy among South African women. Glob Public Health.

[36] Muthuri SK, Oyolola M, Faye C (2017). Trends and correlates of single motherhood in Kenya: results from the Demographic and Health Survey. Health Care Women Int.

[37] Clark S, Dana H (2013). Single motherhood and child mortality in sub-Saharan Africa: a life course perspective. Demography.

[38] Wainaina CW, Wanjohi M, Wekesah F, Woolhead G, Kimani-Murage E (2018). Exploring the experiences of middle income mothers in practicing exclusive breastfeeding in Nairobi. Kenya Matern Child Health J.

[39] Ickes S, Sanders H, Lemein H, Kinyua J, Singa B, McAnally K, King H, Farquhar C, Nduati R, Walson J (2019). Influences of exclusive breastfeeding among low-wage, working mothers in Kenya: perspectives from managers, healthcare providers, daycare directors, mothers, and fathers (OR30-02-19). Curr Dev Nutr.

[40] Kungu W, Khasakhala A, Agwanda A (2020). Trends and factors associated with long-acting reversible contraception in Kenya. F1000Research..

[41] Patel RC, Stalter RM, Thomas KK, Tamraz B, Blue SW, Erikson DW (2019). A pharmacokinetic and pharmacogenetic evaluation of contraceptive implants and antiretroviral therapy among women in Kenya and Uganda. AIDS.

[42] Patel RC, Onono M, Gandhi M, Blat C, Hagey J, Shade SB (2015). Pregnancy rates in HIV-positive women using contraceptives and efavirenz-based or nevirapine-based antiretroviral therapy in Kenya: a retrospective cohort study. Lancet HIV.

[43] McDonough A, Weiser SD, Afkera D, Weke E, Wekesa P, Burger R, Sheira L, Bukusi EA, Cohen CR (2020). “When I Eat Well, I Will Be Healthy, and the Child Will Also Be Healthy”: maternal nutrition among HIV-infected women enrolled in a livelihood intervention in Western Kenya. Curr Dev Nutr.

